# Effect of laser acupuncture on pain, range of motion, and function in patellofemoral pain syndrome: a randomised controlled trial

**DOI:** 10.3389/fmed.2025.1613197

**Published:** 2025-07-15

**Authors:** Nesma M. Allam, Hadeel Alsirhani, Maani Batel Alruwaili, Dalal Mabkhout Dosh, Huriyyah Mislat Alruwaili, Wessal Hisham Almazyad, Rokaia A. Toson, Doaa Ayoub Elimy, Mohamed El-Sherbiny, Ateya Megahed Ibrahim, Hasnaa Ali Ebrahim, Nermine Nosseir, Mohamed A. Eladl, Zeinab A. Ali

**Affiliations:** ^1^Department of Physical Therapy and Health Rehabilitation, College of Applied Medical Sciences, Jouf University, Sakakah, Saudi Arabia; ^2^Department of Physical Therapy for Basic Science, Faculty of Physical Therapy, Cairo University, Cairo, Egypt; ^3^Department of Medical Rehabilitation Sciences, College of Applied Medical Sciences, Najran University, Najran, Saudi Arabia; ^4^Department of Basic Medical Sciences, College of Medicine, AlMaarefa University, Diriyah, Saudi Arabia; ^5^College of Nursing, Prince Sattam Bin Abdulaziz University, Al-Kharj, Saudi Arabia; ^6^Department of Family and Community Health Nursing, Faculty of Nursing, Port Said University, Port Said, Egypt; ^7^Department of Basic Medical Sciences, College of Medicine, Princess Nourah bint Abdulrahman University, Riyadh, Saudi Arabia; ^8^Department of Biomedical Sciences, College of Medicine, Gulf Medical University, Ajman, United Arab Emirates; ^9^Department of Basic Medical Sciences, College of Medicine, University of Sharjah, Sharjah, United Arab Emirates

**Keywords:** laser acupuncture, patellofemoral pain syndrome, low-level laser therapy, anterior knee pain, range of motion, function

## Abstract

**Background:**

Patellofemoral pain syndrome (PFPS) is a knee disorder characterised by pain behind or surrounding the patella that predominantly affects adolescents.

**Objectives:**

This study aimed to evaluate the impact of laser acupuncture (LA) on pain, range of motion (ROM), and function in patients diagnosed with PFPS.

**Materials and methods:**

Sixty participants, predominantly young women aged 18–25 years, were randomly distributed into two equal groups: Group A (LA group), which received laser acupuncture (LA) applied to six knee acupoints at 4 J for 80 s/point, in conjunction with an exercise programme; and Group B (Sham LA group), which received LA with the device deactivated, along with an exercise programme including stretching and strengthening exercises. Both groups underwent the intervention twice a week for 4 weeks. Pain, knee range of motion (ROM), and function were evaluated at baseline and post-treatment using the visual analogue scale (VAS), goniometer, and Kujala score, respectively.

**Results:**

No significant differences were observed in the VAS, ROM, and Kujala scores for knee flexion and extension between the two groups before treatment (*p* > 0.05). However, Group A showed a significant reduction in VAS scores (*p* < 0.001) and a significant improvement in the Kujala score and ROM for knee flexion and extension (*p* < 0.001) compared with Group B.

**Conclusion:**

Four-week LA intervention combined with an exercise programme demonstrated greater improvements in pain, knee ROM, and functional ability than the exercise programme alone. However, the results cannot be generalised, as the sample was mostly composed of young women aged 18 to 25 years.

**Clinical trial registration:**

ClinicalTrials.gov, identifier NCT06610981.

## Introduction

Patellofemoral pain syndrome (PFPS) is one of the most common types of knee discomfort. The primary symptom is usually anterior knee pain, which increases when ascending or descending stairs, bending the knee, jumping, or sitting in a knee-bending position for an extended period (1). PFPS is commonly observed in adolescents and young adults, with a prevalence rate that is twice as high in women as in men. The prevalence of PFPS in the general population is 22.7, and 28.9% in teenagers (2). Pain worsens with knee joint movement, limiting daily activities such as climbing stairs and kneeling (3).

To better understand the underlying causes of PFPS, it is important to consider known risk factors. These include tightness in the hamstring, gastrocnemius, and iliotibial bands, as well as muscle imbalances identified in functional tests, particularly involving the quadriceps, vastus medialis obliquus (VMO), hamstring, and hip muscles. Additionally, increased quadriceps (Q) angle and patellar displacement are contributing factors (4, 5). An unbalanced muscular pull on the patella or VMO weakness may be the reason for inappropriate patellar positioning, resulting in excessive lateral movement of the patella (6).

Various conservative treatment approaches have been employed to manage PFPS, including physiotherapy, chiropractic therapy, and traditional Chinese medicine (7). Several studies have shown that strengthening both the hip abductor and quadriceps muscles results in greater pain relief and an enhancement of knee function compared with limited training of the quadriceps muscles, which would reduce the force exerted on the patellofemoral joint (6, 8). Other conservative methods include knee braces, patellar banding, electrophysical modalities (such as neuromuscular electrical stimulation, biofeedback, transcutaneous electrical nerve stimulation (TENS), thermotherapy, and ultrasound), strengthening exercises for the gluteal muscles, hamstring, and tibialis anterior, stretching exercises for the lateral retinaculum and iliotibial band, changing activities, and wearing suitable shoes (9).

Among the emerging treatment modalities, low-level laser therapy (LLLT) has attracted attention for its reparative properties and non-thermal effects on human cartilage using a monochromatic light source that does not generate heat. Additionally, it exhibits pain-relieving effects; however, the underlying mechanisms remain unclear. Hypothesised explanations include enhanced mitochondrial adenosine triphosphate (ATP) production and tissue oxygenation, improved release of neurotransmitters associated with pain regulation (such as serotonin), and the anti-inflammatory properties of the laser (10, 11).

Laser acupuncture (LA) is a non-invasive technique that uses laser energy to activate acupuncture points and has the benefits of traditional acupuncture, such as decreasing pain, swelling, and inflammation (12), enhancing local metabolism (13), improving blood circulation in the affected areas, accelerating tissue regeneration, and reducing muscle tension around the knee joint (14). It stimulates the pituitary gland to release endorphins that relieve pain and improve knee range of motion (ROM), thus improving overall knee function and allowing patients to return to their normal activities more quickly (15).

Although the benefits of LA have been explored in several musculoskeletal disorders, such as knee osteoarthritis (10), rheumatoid arthritis (16), temporomandibular disorders (17), and lower back pain (18), no previous studies have evaluated the impact of LLLT on acupoints in pain management, knee ROM, and function in patients with PFPS. The scientific contribution of this study lies in its potential to establish LA as a safe, non-pharmacological, and minimally invasive treatment option that can alleviate pain, enhance knee ROM, and improve the function of patients with PFPS. By integrating two evidence-supported modalities (LLLT and acupuncture point stimulation), this study explored a synergistic intervention that may decrease reliance on non-steroidal anti-inflammatory drugs (NSAIDs) and invasive procedures, thereby broadening the spectrum of conservative management strategies for PFPS. This study not only addresses a current gap in the literature but also has the potential to establish foundational protocols for future applications in physical therapy and rehabilitation. It offers clinicians a novel tool for enhancing outcomes in patients with PFPS. Therefore, the aim of the present study was to examine the effects of LA on pain alleviation and improvements in knee ROM and function in patients with PFPS.

## Materials and methods

### Study design

The current study was a prospective, randomised, double-blind, sham-controlled trial conducted at the Physical Therapy Laboratory, College of Applied Medical Sciences, Jouf University, from October 2024 to May 2025. All study procedures were carried out after obtaining approval from the Institutional Review Board of the Hail Health Cluster, KSA (IRB log no. 2024–82). This study was registered at ClinicalTrials.gov (NCT06610981). Before beginning the study, all study details were explained to the participants, and they were asked to sign a written informed consent form. This study adhered to the CONSORT standards.

### Subjects

Sixty participants were selected for inclusion in the study based on the following criteria: individuals of both genders aged 18–25 years. Participants diagnosed with PFPS according to the International Classification of Diseases (ICD), specifically using the M22.2 × 9 code, who demonstrated positive functional test results and normal knee radiographs, were included (19). Participants with positive functional tests performed by the physician, including manual compression of the patella over the femur during rest or isometric quadriceps contraction, patella palpation, isometric quadriceps contraction against resistance, squatting with both legs, kneeling, and climbing stairs, were included. A pain score of >3 cm on the visual analogue scale (VAS), located at the anterior, retro-patellar, or peri-patellar aspects of the knee, which was exacerbated by repetitive activities and alleviated by rest for at least 3 months, was also an inclusion criterion. Participants with normal knee joint radiographs in all dimensions (patellar view, A-P, and lateral) and who were able to follow the treatment guidelines were included. The exclusion criteria included participants with BMI ≥ 30, physiotherapy, chiropractic treatment, or massage therapy in the last 12 months; injection in the knee joint in the last 3 months; other knee disorders, such as osteoarthritis, previous knee surgery, meniscal injury, recent knee trauma, joint effusion, patellar subluxation/dislocation, intra-articular derangement, patellar tendonitis, joint deformity, or bursitis; pregnancy, pacemaker insertion, photosensitivity, and receiving NSAIDs in the previous 4 weeks; history of malignancy, infection, psychiatric disorder, intellectual disability, autoimmune diseases, neurologic dysfunction that might affect walking; and history of chronic diseases, such as diabetes mellitus and uncontrolled hypertension. The participants were randomly assigned to two groups: Group A (LA group) received LA in combination with a traditional physical therapy programme twice a week for 4 weeks, while Group B (Sham LA group) underwent LA with the device deactivated, alongside a traditional physical therapy programme twice a week for the same period. The measurements were carried out at baseline and at 4 weeks following the treatment programme for group A and group B.

### Sample size

The sample size was calculated using G*Power software (version 3.1.9.2; Heinrich-Heine-Universität, Düsseldorf, Germany) according to the changes in the primary outcome (pain score assessed using VAS). The effect size for the LA and sham LA groups was estimated based on the means and standard deviation within groups, as reported by Nouri et al. (20), who used a similar intervention (laser therapy) for PFPS, ensuring that the assumed variability reflected the clinically relevant data. An effect size of 0.72 was applied at 80% power to detect effect sizes with *α* = 5%. According to these assumptions, the estimated sample size for the t-test was 25 participants per group (50 participants in total). The dropout rate was calculated as 20% to ensure that the final number of participants completing the study was sufficient to maintain adequate statistical power. The final sample comprised 60 participants, with 30 individuals allocated to each group.

### Randomisation

Using a computer-based tool at http://www.randomization.com/, block randomisation was carried out by an independent researcher not included in the examination or treatment processes. To avoid bias and differences between the groups, 60 participants who fulfilled the inclusion criteria were randomly assigned to either group A (LA group) or group B (Sham LA group) in a 1:1 ratio (30 participants in each group), and the randomisation depended on blocks of four.

### Blinding

Neither the participants nor the examiner were aware of whether the LA or sham LA group was being administered; only the independent researcher possessed the identifying code to detect which therapy was being utilised. Participant blindness was encouraged by using the same laser instrument with invisible laser radiation without heat or sound for both groups at the same time and location. The statistician was blinded to the treatment assignments until analyses were completed.

Following the last therapy session, a structured post-intervention questionnaire was administered to assess the efficacy of the participant blinding. Participants were asked if they thought they had received active or sham laser therapy, and their responses were categorised as “correct,” “incorrect,” or “unsure.” Their responses were qualitatively analysed to determine whether participants could accurately identify their group assignment or whether their expectations regarding the treatment or procedural signals influenced them.

### Procedures

Before participating in the study, the participants underwent an initial interview, followed by an orthopaedic knee examination. This process aimed to identify any abnormalities, such as congenital deformities including genu valgum, genu varum, or knee dislocation, through physical examination and radiographic assessment. Additionally, neurological impairments that could potentially affect study outcomes, such as hypoesthesia, paraesthesia, or neuropathic pain, were evaluated using a comprehensive neurological examination, including sensory, motor, reflex, and neurodynamic tests (21). Additionally, the stability of the knee ligaments was assessed using stress tests as described by Konin et al. (22).

### Intervention

Following the completion of the baseline measurements, the third author opened sealed envelopes containing the participants’ group assignments and administered the treatment accordingly. All participants underwent two familiarisation sessions to review the prescribed training and to receive instructions on proper exercise techniques. Subsequently, the participants were randomly assigned to the LA or Sham LA groups.

### Traditional physical therapy programme

Participants in the LA and Sham LA groups underwent a traditional physical therapy programme. The physical therapy programme included strengthening exercises, such as isometric quadriceps exercises, isometric hip adduction, straight leg raising (upward and laterally), squatting to 30° knee flexion, and stretching exercises for the hamstring, iliotibial band, and gastrocnemius (23, 24), as detailed in Supplementary material 1. The same physiotherapist performed the exercises on all participants. The exercises were performed in three sets of 10 repetitions per day, with a 2-min break between sets, for a total of 20–30 min throughout the course of the treatment (25).

### Laser acupuncture

The room was maintained at a constant temperature of 20°C throughout the treatment period. The participants and the researcher wore eye goggles to protect against laser radiation, which helped the participants to be masked from the intervention. The participants were instructed to maintain a supine position with an extended knee (resting position of the patellofemoral joint), and the acupuncture points were selected and sterilised using an alcohol-based swab (25). LA was performed using a low-level diode laser pen (peak power, 100 W; pulse duration, 150 ns; wavelength, 905 nm; power output, 100 mW; and pulsed wave; Endolaser-442, Enraf Nonius, Rotterdam, The Netherlands). The laser pen, with invisible laser radiation, was positioned in a perpendicular direction over six points (ST34, ST 35, GB34, EX-LE4, SP9, and SP10), which were detected based on previous studies (15, 26) (Table 1). Each point was supplied with 4 J for 80 s, with a total of 24 J per session (27). Each LA treatment was performed by a skilled physiotherapist with >7 years of experience and training. In group A (LA group), the laser device was turned on for 8 min; in group B (Sham LA group), the laser device was turned off and applied to the acupoints for the same period. The treatment was repeated twice weekly for 4 weeks.

**Table 1:** Location of acupuncture points used in the current study.

Acupuncture point	Location
ST34 (Liangqiu)	Located at the top of the patella’s laterosuperior margin.
ST 35 (Dubi)	Suited in the groove of the lateral aspect of patella as well as the patellar ligament.
GB34	Suited in the groove inferior and anterior to the fibular head on the lateral surface of the leg
EX-LE4	Found in the depression medial to the patellar ligament when the knee is flexed.
SP9 (Yinlingquan)	Found at the medial aspect of the knee on the sural nerve, that is related to pain in the knee joint.
SP 10 (Xuehai)	Suited medially in the thigh, with knee flexion, 2 cun over the patella’s mediosuperior margin, medially on the bulk of the quadriceps muscle.

### Outcome measures

Instructions were provided to the participants on how to complete the VAS and KUJALA questionnaires, examinations were conducted, and preliminary data, such as age, height, weight, body mass index (BMI), and duration of symptoms, were gathered. A dominant painful knee was selected if the participants had symptoms in both knees or if the symptoms were the same on both sides. The primary outcome measure was pain intensity, which was assessed using the VAS score. The secondary outcome measures included the ROM of knee flexion and extension measured using a universal goniometer and the function of the knee joint evaluated using the KUJALA scale. All outcome measures were recorded at the beginning of the study and after 4 weeks.

#### Primary outcome measure

##### Pain assessment

Pain intensity was assessed using the VAS, a 10 cm horizontal line, where 0 cm represents the worst possible pain. Participants were asked to mark their current pain levels on the line. The VAS score was recorded at baseline and 4 weeks after the intervention. VAS is widely used in clinics and research because it has been demonstrated to be a valid and reliable method for evaluating pain (28).

#### Secondary outcome measures

##### Knee flexion and extension ROM

Knee flexion and extension ROM were measured using a universal goniometer, which is a valid and reliable instrument that demonstrates excellent inter-rater reliability [intraclass correlation coefficient (ICC) = 0.996]. The participants lay prone with their knee in extension to measure passive knee flexion and were instructed to move the heel of the affected leg towards the buttock as closely as possible while maintaining contact with the other foot on the bed. The fulcrum of the goniometer was aligned with the femoral lateral epicondyle of the affected knee, with the stationary arm oriented towards the greater trochanter and the moving arm directed towards the lateral malleolus. A ROM of 135° was considered normal for knee flexion (29).

To measure extension ROM, the participants were instructed to lie in a supine position, and the hip and knee of the affected limb were flexed to 90°, with the foot in a comfortable position. Two straps secured a wooden box to the bed, a third held the thigh of the participant with the box, and a fourth was placed around the thigh of the opposite limb to reduce hip flexion during the measurement. The universal goniometer was used for measurement of knee extension angle, which has a high inter-rater reliability (ICC = 0.996) (30). The fulcrum of the universal goniometer was then applied to the lateral epicondyle of the distal femur, with the stationary arm directed towards the greater trochanter, while the moving arm was directed towards the lateral malleolus. Subsequently, the first evaluator passively and gradually moved the knee in extension, whereas the second evaluator confirmed that no substitutions had occurred. During the examination, the participants were asked to relax, particularly with their knee in the extended position, and to identify the point at which they noticed the beginning of tension in the knee flexors, which was detected as the end of the range. After reaching this position, the participant maintained it for 5 s to enable measurement of the knee extension angle. Full knee extension was determined to be 0° and was used to detect the restriction of knee extension.

Three measurements were taken for knee flexion and extension ROM for each movement, with a 1-min rest in between, and the average was used for analysis. To ensure reliable data, all measurements were recorded at baseline and after 4 weeks of intervention using the same goniometer by the same individual (30, 31).

##### Knee function

The anterior knee pain (Kujala) score is used to assess knee function in individuals with PFPS. This grading system assigns values ranging from 100, indicating normal knee function without pain, to 0, indicating severe knee pain and dysfunction. Haddad et al. (32) examined the validity and reliability of the Arabic version of the Kujala scale.

#### Adverse events and safety monitoring

The safety of LA was closely monitored throughout the study to ensure the wellbeing of the participants and assess any potential adverse effects. The side effects of laser therapy were evaluated based on a combination of self-reported symptoms, clinical observations, and medical history, including headaches, burns, temporary dizziness, skin erythema, and fatigue (33). Before enrolment, the participants were informed about possible adverse effects and asked to notify the research staff immediately if they experienced any abnormal symptoms. The type, intensity, onset, and duration of any reported symptoms were recorded using standardised adverse event reporting forms. The forms were routinely evaluated by a primary investigator. All participants were evaluated for indications of discomfort or negative reactions both before and after each session, as part of the safety monitoring routine. Vital signs were monitored regularly to ascertain physiological stability.

As a stopping rule, any participant who experienced a serious or ongoing adverse event based on the professional judgement of the study clinician was temporarily withdrawn from the intervention until the necessary evaluation was completed. Participants were excluded from the trial if the adverse events were found to be substantial and associated with the intervention.

### Statistical analysis

Statistical analyses were performed using the SPSS software (version 25.0; IMB Corp., Armonk, NY, United States). An independent t-test was used to compare the characteristics of the participants across different groups. The chi-square test was used to assess the distribution of the most affected side and sex between the groups. The Shapiro–Wilk test was used to confirm the normality of the data distribution. Levene’s test for homogeneity of variance was used to evaluate the group homogeneity. The effects on the VAS, Kujala score, and knee flexion and extension ROM were compared between and within groups using a mixed-design MANOVA. To ensure multiple comparisons, post-hoc analyses were conducted using the Bonferroni correction. The significance level for all statistical tests was set at *p* ≤ 0.05.

## Results

### Demographic and subject characteristics

**Figure 1:**
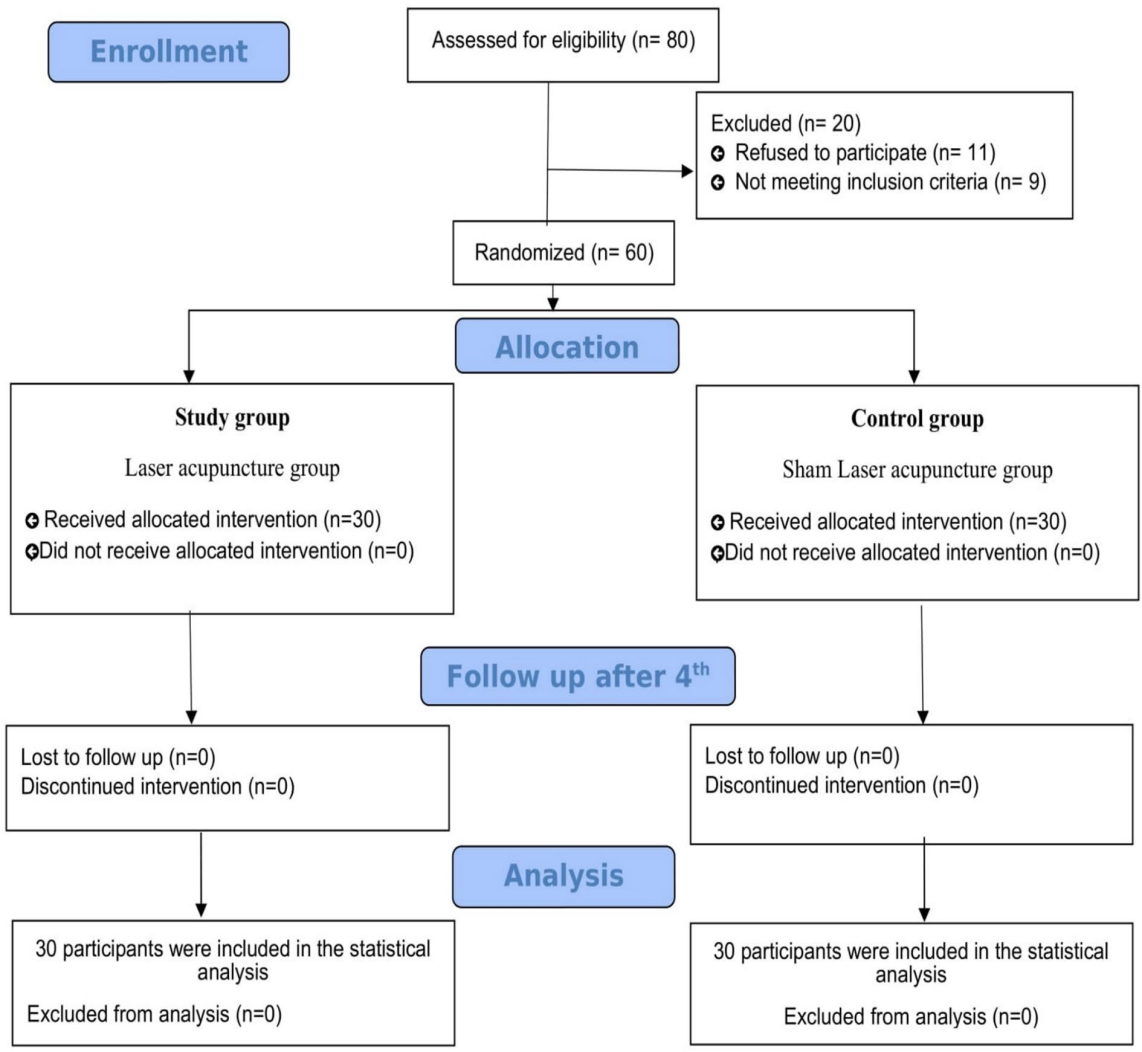
Flowchart of the laser acupuncture clinical study of patellofemoral pain syndrome.

A total of 60 participants completed the study. Figure 1 presents a flowchart detailing the selection process for participants in the current study. Initially, 80 individuals were selected, but 20 were excluded. Of these, nine did not meet the inclusion criteria and 11 declined to participate. The remaining 60 participants were randomly allocated to the LA and Sham LA groups, with 30 participants per group. All participants completed the intervention and were included in the statistical analyses.

The characteristics of all the participants in both groups are presented in Table 2. There were no significant differences between the two groups in terms of age, weight, height, BMI, duration of symptoms, and gender, indicating successful randomisation between the two groups (*p* > 0.05).

**Table 2:** Subject characteristics.

Variables	Group A	Group B	MD	t-value	*p*-value
	Mean ± SD	Mean ± SD			
Age (years)	21.43 ± 1.85	20.83 ± 1.74	0.60	1.29	0.20
Weight (kg)	64.00 ± 6.69	63.73 ± 7.00	0.27	0.15	0.88
Height (cm)	165.33 ± 5.76	164.80 ± 4.51	0.53	0.39	0.69
BMI (kg/m^2^)	23.42 ± 2.08	23.37 ± 2.11	0.05	0.09	0.92
Duration of symptoms (months)	13.07 ± 6.21	12.73 ± 5.35	0.34	0.22	0.83
Sex, *n* (%)					
Females	21 (70%)	20 (67%)		χ^2^ = 0.08	0.78
Males	9 (30%)	10 (33%)			
Affected side, *n* (%)					
Right	15 (50%)	16 (53%)			
Left	11 (37%)	9 (30%)		χ^2^ = 0.34	0.84
Bilateral	4 (13%)	5 (17%)			

SD, standard deviation; MD, mean difference; χ^2^, Chi-squared value; *p*-value, level of significance.

### Effect of treatment on VAS, Kujala score, flexion, and extension ROM

The interaction between the treatment and time was statistically significant (*F* = 99.17, *p* = 0.001, η^2^ = 0.88), indicating that the treatment effect varied over time. Additionally, the main effect of time was significant (*F* = 326.76, *p* = 0.001, η^2^ = 0.96), demonstrating that outcomes changed significantly over time across all groups. The main effect of treatment (*F* = 12.16, *p* = 0.001, η^2^ = 0.47) revealed significant differences between the groups. Consequently, the η^2^ values observed in this study exceeded 0.14, indicating large effect sizes and suggesting that both time and treatment had a substantial impact on pain, knee ROM, and function in patients with PFPS.

Within-group comparisons showed that the VAS score significantly decreased and the Kujala score significantly increased in groups A and B post-treatment compared with pre-treatment (*p* > 0.001) (Table 3). A significant increase was found in the knee ROM for flexion and extension in groups A and B compared to the pre-treatment values (*p* > 0.001) (Table 4).

**Table 3:** Mean VAS and Kujala scores pre- and post-treatment of groups A and B.

Variables	Group A	Group B	MD (95% CI)	*p* value	ES
	Mean ±SD	Mean ±SD			
VAS					
Pre treatment	6.60 ± 1.85	6.40 ± 1.73	0.2 (−0.73: 1.13)	0.67	
Post treatment	1.53 ± 1.14	5.27 ± 1.72	−3.74 (−4.49: −2.98)	0.001	2.56
MD (95% CI)	5.07 (4.69: 5.44)	1.13 (0.76: 1.51)			
% of change	76.82	17.66			
	*p* = 0.001	*p* = 0.001			
Kujala score					
Pre treatment	69.80 ± 8.66	69.27 ± 9.83	0.53 (−4.25: 5.32)	0.82	
Post treatment	89.13 ± 7.81	78.10 ± 10.37	11.03 (6.29: 15.78)	0.001	1.20
MD (95% CI)	−19.33 (−21.44: −17.22)	−8.83 (−10.94: −6.72)			
% of change	27.69	12.75			
	*p* = 0.001	*p* = 0.001			

SD, Standard deviation; MD, Mean difference; CI, Confidence interval; *p*-value, Level of significance; ES, Effect size.

**Table 4:** Mean flexion and extension ROM pre- and post-treatment of groups A and B.

ROM (degrees)	Group A	Group B	MD (95% CI)	*p* value	ES
	Mean ± SD	Mean ± SD			
Flexion					
Pre treatment	115.60 ± 7.41	114.43 ± 10.04	1.17 (−3.39: 5.73)	0.61	
Post treatment	133.03 ± 4.32	120.10 ± 10.05	12.93 (8.94: 16.93)	0.001	1.67
MD (95% CI)	−17.43 (−19.09: −15.78)	−5.67 (−7.32: −4.01)			
% of change	15.08	4.95			
	*p =* 0.001	*p =* 0.001			
Extension					
Pre treatment	37.10 ± 12.17	36.77 ± 9.21	0.33 (−5.24: 5.91)	0.91	
Post treatment	13.77 ± 7.45	30.53 ± 9.22	−16.76 (−21.10: −12.43)	0.001	2
MD (95% CI)	23.33 (21.06: 25.61)	6.24 (3.96: 8.51)			
% of change	62.88	16.97			
	*p =* 0.001	*p =* 0.001			

SD, Standard deviation; MD, Mean difference; CI, Confidence interval; *p*-value, Level of significance; ES, Effect size.

However, no significant differences were observed between the groups before treatment (*p* > 0.05). Post-treatment analysis indicated a significant reduction in the VAS (ES = 2.56) and a notable increase in the Kujala score (ES = 1.20), flexion ROM (ES = 1.67), and extension ROM (ES = 2) in Group A compared to those in Group B (*p* < 0.001). Consequently, all observed effect sizes in this study exceeded 0.8, signifying large treatment effects for all the variables (Tables 3, 4).

### Blinding effectiveness

To evaluate the blinding effectiveness of the participants, the Bang’s Blinding Index (BI) was calculated for the LA and Sham LA groups. In the LA group (*n* = 30), six participants successfully guessed the group to which they were allocated, 13 provided an inaccurate estimate, and 11 were uncertain, obtaining a BI of −0.184. The Sham LA group (*n* = 30) had a BI of −0.25, with five participants accurately predicting their allocation, 15 making a wrong guess, and 10 being unsure. These results indicate that the participants were more likely to guess incorrectly than correctly, as the BI values were negative and close to 0, suggesting that blinding was effective in both groups.

### Adverse effects

However, no major adverse effects were observed throughout the study, and no participants were permanently excluded. Following the first session, three participants in the LA group experienced mild and temporary adverse effects, such as skin redness with a 2-mm diameter at the laser radiation location. Erythema disappeared after a few hours and did not require treatment. No additional side effects were observed after the treatment.

## Discussion

Patellofemoral pain syndrome frequently occurs in a wider community, particularly in young active adults and adolescents, although it is most common in women. Owing to its high incidence (15–45%) and long-lasting complaints, it places significant financial pressure on healthcare systems (34, 35). This study investigated the efficacy of laser acupuncture combined with an exercise programme on pain, ROM for knee flexion and extension, and function in patients with patellofemoral pain syndrome (PFPS).

The laser dosage applied in this study was determined in accordance with the World Association for Laser Therapy (WALT) guidelines, which recommend a laser dose of ≥1 J for each treatment point on the knee joint line or synovium, utilising a mean power of 5–500 mW and a wavelength of 905 nm (36). Furthermore, the therapeutic reference for joint treatment is specified as 3–8 J per acupoint, based on recommendations for Low-Level Laser Therapy (LLLT) practice (37). These acupoints were selected because of their efficacy in enhancing blood circulation; tonifying Qi; relaxing muscles and tendons; eliminating harmful wastes; removing dampness; regulating channels; alleviating tightness, weakness, or paralysis of the lower limb muscles; relieving knee joint pain; increasing blood supply around the knee; reducing swelling and soreness; and improving joint mobility (38). In traditional Chinese medicine, these points are most frequently used to treat knee disorders (10).

Following the intervention, both the LA and sham LA groups showed significant improvements in pain, ROM, and function within their respective groups; however, the LA group demonstrated significantly better outcomes than the sham LA group (*p* < 0.001). These findings indicate that the addition of laser acupuncture provides additional benefits to patients with PFPS.

These results exceeded the minimal clinically important difference (MCID) for the Visual Analogue Scale (VAS), which was 2 cm for individuals with patellofemoral pain syndrome (PFPS) (39). The MCID denotes the smallest change in the outcome of any intervention perceived by patients to be beneficial, thereby justifying a modification in patient management (40). These findings suggest that local anaesthetics (LA) result in a significant clinical improvement in the pain threshold, thereby supporting their efficacy in reducing pain in a clinically meaningful manner.

The pain-relieving effects of LA may be attributed to LA’s capacity to interfere with afferent signals to the central nervous system (fibres C and A) due to increased activity of the Na+-K+pump, which subsequently elevates pain intensity and reduces joint pain and muscle spasms. Additionally, LA enhances the regeneration of *β*-endorphins, which are neurotransmitters involved in pain modulation, within the synovial membrane, along with protein production (31). Furthermore, LA exhibits anti-inflammatory effects that mitigate pain by reducing the levels of metalloproteinase 3, pro-inflammatory cytokines IL-6, IL-1b, prostaglandin E2, tumour necrosis factor *α*, and other enzymes (11). It also increases collagen II, growth factor beta, and aggrecan levels, thereby promoting cartilage regeneration and elevating serum levels of nitric oxide, leading to vasodilation and improved blood circulation in the knee joint (33).

Consistent with our results, several studies have reported significant improvements in VAS scores after LA administration in patients with KOA (10, 27, 41–46). A significant improvement was recorded in the VAS scores in the LA group compared with the placebo group after LA was applied to the knee acupoints (ST35, ST36, GB34, SP9, SP10, and EX-LE-4). This result is supported by the elevation of endogenous opioid and anti-inflammatory cytokine levels (10, 27, 44–46). Short-term application of LA to acupuncture points (EX-LE4, EX-LE5, ST 35, ST 36, GB 34, SP 9, and SP 10) in association with quadriceps strengthening and stretching exercises significantly relieved the pain. These findings support the use of LA as a valuable supplemental treatment for the knee joint, and potentially for various joints (41–43).

However, contrasting results have been reported in the literature. One study reported a non-significant effect on pain and function in patients with KOA (47). This contradiction may be attributed to the shorter duration of application (2 weeks), lower dose (0.48 J), and low power density (10 mW/cm2), which may have been insufficient to stimulate the acupuncture point through the activation of mast cell degranulation (27). Additionally, the application of LA is limited to a single acupuncture point (SP 9).

In terms of ROM, LA caused a significant improvement in knee ROM (*p* < 0.001) in the study group compared with the sham group after 4 weeks. Unfortunately, no previous study has detected the MCID for knee ROM in patients with PFPS. However, the MCID for knee flexion in patients with KOA was 3.8°–6.4° (48) and 7°–8° for knee flexion and extension ROM in stroke patients (49), and the results of the present study were higher than the MCID for these studies. This indicates that LA surpasses the minimum threshold for clinically significant enhancement of knee range of motion (ROM). This suggests that the change is not only statistically significant but also represents a clinical improvement in joint mobility that patients can perceive in daily activities, such as climbing stairs and walking. It has been reported that LLLT increases knee ROM by improving collagen synthesis by enhancing the amount of hydroxyproline throughout the build-up of ascorbic acid in fibroblasts. Furthermore, LLLT improves the number of fibroblasts and epithelial cells by promoting blood circulation and oxygenation, which are necessary for enhancing the cellular activities required for tissue healing (50, 51). This stimulation helps to restore normal joint function and flexibility (52). The analgesic effect of LLLT enhances participation in physical therapy exercises, which can further improve ROM (53). The photobiomodulatory effects of laser light stimulate mitochondrial activity, leading to increased adenosine triphosphate (ATP) production. This increase in energy supports the cellular functions necessary for healing and regeneration, which play roles in improving joint mobility (54).

Consistent with previous studies, the study found a significant increase in knee ROM after LA in patients with KOA (44, 52, 55). A significant improvement in knee flexion ROM was recorded after the application of LA on acupoints (ST 36, ST 35, GB 34, Sp 9, Sp 10, Sp 6, and liv3) (44), after combining LA on knee acupoints with knee exercises (55), and after the addition of LLL therapy to an intensive functional rehabilitation programme in patients with knee replacement (52).

Regarding knee function, the current study showed that LA had a significant effect on enhancing knee function in the LA group when compared with the sham group (*p* < 0.001), which was greater than the MCID for the Kujala scale (10 points) in patients with PFPS (39). The findings suggest that LA’s impact is both statistically significant and clinically important, as participants are expected to see noticeable and beneficial improvements in overall knee function, confirming LA as a viable therapeutic option. LA enhances function by improving collagen production and biostimulation, and accelerating the healing process by up to 40% by reinforcing fibrous tissue and collagen production (56). LA maintains muscle performance by promoting the growth and replication of myosatellite cells and improving angiogenesis and myotube synthesis by boosting muscle fibre regeneration and mitochondrial density (57). Additionally, exercise performance was enhanced by the analgesia provided by the LA, and this combination led to greater muscle function. Furthermore, this enhancement might be related to LA’s capacity of LA to produce effects similar to those of acupuncture needles at the skin level through an inhibitory mechanism throughout the neural blockade (58).

In line with the findings of the current study, previous studies have reported improved knee function after LA in patients with KOA (10, 27, 42–45, 55, 58–60). LA was reported to be beneficial in enhancing functional ability when applied to acupuncture points (ST35, SP9, SP10, ST36, GB34, and EX-LE 4) (10, 27, 44, 58, 60), as well as reducing stiffness early after total knee replacement in the first three postoperative days (55). Additionally, the application of LA at acupuncture points (ST34, GB34, ST35, EX-LE4, SP10, and SP9) with stretching and strengthening exercises enhanced the functional state. Therefore, LA may be suggested in intervention guidelines (42, 43, 45). Moreover, LA was superior in improving functional ability and alleviating morning stiffness compared with acupuncture therapy alone. Thus, LA results in better clinical outcomes in terms of physical function scores (59).

Interestingly, a non-significant effect of high-intensity laser therapy on pain and function was reported in patients with KOA. This might be explained by the different types of lasers used in this study and different application techniques (61).

The enhancements shown in the sham LA group may be related to various factors, such as the impact of the sham, and all participants in both groups performed exercises frequently and were better informed about their medical state. Participants with PFPS responded well to the exercise programme, which is consistent with the literature (20). Training of the hip abductor and quadriceps muscles decreased the force in the patellofemoral joint, decreased pain intensity, and improved functional ability because of increased coordination of the lower extremity muscles, which is in accordance with other studies (8, 62). Additionally, sensory adaptation is a key mechanism that influences the effect of stretching on ROM. Thus, stretching exercises to improve knee ROM and functional status should be part of any successful training programme (63).

### Strengths and limitations

One of the strengths of this study is its randomised, double-blind, and sham-controlled design, which minimises bias and enhances the internal validity of the findings. The use of a standardised exercise programme in both groups allowed for a clear evaluation of the additional benefits of LA. Furthermore, the inclusion of objective outcome measures (VAS, goniometer, and Kujala scores) strengthens the reliability of the results. However, this study has several limitations. The sample was predominantly composed of 18–25 year old women, and they were selected from only one centre, which limits the generalisability of the findings to other populations, such as men or different age groups. Future research should include a more diverse participant pool to assess the efficacy of LA in a broader demographic population. Additionally, the relatively short 4-week intervention period may not be sufficient to observe long-term effects. Future studies should investigate the long-term benefits of LA in PFPS. The study also did not include a follow-up period to assess the sustained effects of interventions.

Although the diagnosis in this study was based on radiographic imaging and clinical assessment, the lack of sonographic or MRI confirmation could have lowered the diagnostic specificity. In the absence of comprehensive imaging, minor structural anomalies or differential diagnoses can go unnoticed, which could compromise the precision of participant selection and treatment effect interpretation. Therefore, future studies should include sonographic or MRI examinations to improve the diagnostic accuracy and fortify the reliability of clinical evaluations. Moreover, further research is recommended to examine how incorporating LA into tele-rehabilitation models or digital health platforms could increase access, enhance adherence, and provide individualised care for patients with PFPS living in rural or underserved areas.

## Conclusion

Laser acupuncture applied to knee acupoints significantly reduced pain, improved knee ROM (flexion and extension), and enhanced knee function in patients with PFPS. Clinically, LA provides a non-invasive and safe method to significantly induce pain relief and restore knee function and mobility, which can encourage more patients to participate in rehabilitation programmes. Combining LA with stretching and strengthening exercises can accelerate recovery and reduce reliance on medication. However, further research with diverse populations and longer follow-up periods is warranted to confirm these findings and to explore the long-term efficacy of this combined approach.

## Data Availability

The original contributions presented in the study are included in the article/Supplementary material, further inquiries can be directed to the corresponding author.

## Supplementary material

The Supplementary material for this article can be found online at: https://www.frontiersin.org/articles/10.3389/fmed.2025.1613197/full#supplementary-material

SUPPLEMENTARY TABLE S1 Description of traditional physical therapy program used in the study.

## References

[1] Sisk D Fredericson M. Update of risk factors, diagnosis, and Management of Patellofemoral Pain. Curr Rev Musculoskelet Med. (2019) 12:534–41. doi: 10.1007/s12178-019-09593-z, PMID: 31773479 PMC6942114

[2] Smith BE Selfe J Thacker D Hendrick P Bateman M Moffatt F. Incidence and prevalence of patellofemoral pain: a systematic review and meta-analysis. PLoS One. (2018) 13:e0190892. doi: 10.1371/journal.pone.0190892 29324820 PMC5764329

[3] Culvenor AG Segal NA Guermazi A Roemer F Felson DT Nevitt MC. Sex-specific influence of quadriceps weakness on worsening patellofemoral and tibiofemoral cartilage damage: a prospective cohort study. Arthritis Care Res. (2019) 71:1360–5. doi: 10.1002/acr.23773, PMID: 30295439 PMC6453735

[4] Hu H Zheng Y Liu X Gong D Chen C Wang Y. Effects of neuromuscular training on pain intensity and self-reported functionality for patellofemoral pain syndrome in runners: study protocol for a randomized controlled clinical trial. Trials. (2019) 20:409. doi: 10.1186/s13063-019-3503-4, PMID: 31288849 PMC6617607

[5] Alvim FC Muniz AMDS Lucareli PRG Menegaldo LL. Kinematics and muscle forces in women with patellofemoral pain during the propulsion phase of the single leg triple hop test. Gait Posture. (2019) 73:108–15. doi: 10.1016/j.gaitpost.2019.07.193, PMID: 31323618

[6] Nascimento LR Teixeira-Salmela LF Souza RB Resende RA. Hip and knee strengthening is more effective than knee strengthening alone for reducing pain and improving activity in individuals with patellofemoral pain: a systematic review with Meta-analysis. J Orthop Sports Phys Ther. (2018) 48:19–31. doi: 10.2519/jospt.2018.7365, PMID: 29034800

[7] Chun-Pu Chu E Fu Chieh Lin A. Knee pain following total knee arthroplasty secondary to cervical spondylotic myelopathy. Curr Health Sci J. (2022) 48:226–9. doi: 10.12865/CHSJ.48.02.13 36320881 PMC9590355

[8] Alammari A Spence N Narayan A Karnad SD Ottayil ZC. Effect of hip abductors and lateral rotators’ muscle strengthening on pain and functional outcome in adult patients with patellofemoral pain: a systematic review and meta-analysis. J Back Musculoskelet Rehabil. (2023) 36:35–60. doi: 10.3233/BMR-220017, PMID: 35988215

[9] Kuwabara A Cinque M Ray T Sherman SL. Treatment options for patellofemoral arthritis. Curr Rev Musculoskelet Med. (2022) 15:90–106. doi: 10.1007/s12178-022-09740-z, PMID: 35118631 PMC9083346

[10] Liao FY Lin CL Lo SF Chang CC Liao WY Chou LW. Efficacy of acupoints dual-frequency low-level laser therapy on knee osteoarthritis. Evid Based Complement Alternat Med. (2020) 2020:6979105. doi: 10.1155/2020/6979105 33029170 PMC7532399

[11] Tomazoni SS Leal-Junior ECP Pallotta RC Teixeira S De Almeida P Lopes-Martins RÁB. Effects of photobiomodulation therapy, pharmacological therapy, and physical exercise as single and/or combined treatment on the inflammatory response induced by experimental osteoarthritis. Lasers Med Sci. (2017) 32:101–8. doi: 10.1007/s10103-016-2091-8, PMID: 27726040

[12] De Oliveira MF Johnson DS Demchak T Tomazoni SS Leal-Junior EC. Low-intensity LASER and LED (photobiomodulation therapy) for pain control of the most common musculoskeletal conditions. Eur J Phys Rehabil Med. (2022) 58:282. doi: 10.23736/S1973-9087.21.07236-1, PMID: 34913330 PMC9980499

[13] Matos LC Machado JP Monteiro FJ Greten HJ. Understanding traditional Chinese medicine therapeutics: An overview of the basics and clinical applications. Healthcare. (2021) 9:257. doi: 10.3390/healthcare9030257, PMID: 33804485 PMC8000828

[14] Dusek JA Kallenberg GA Hughes RM Storrow AB Coyne CJ Vago DR. Acupuncture in the emergency department for pain management: a BraveNet multi-center feasibility study. Medicine (Baltimore). (2022) 101:e28961. doi: 10.1097/MD.0000000000028961, PMID: 35244059 PMC8896475

[15] Wen X Zhang G Cui J Tang Y Meng Q Su Y. Efficacy and safety of laser acupuncture on osteoarthritis: a systematic review and meta-analysis. Front Aging Neurosci. (2025) 16:1462411. doi: 10.3389/fnagi.2024.1462411, PMID: 39845448 PMC11751068

[16] Adly AS Adly AS Adly MS. Effects of laser acupuncture tele-therapy for rheumatoid arthritis elderly patients. Lasers Med Sci. (2022) 37:499–504. doi: 10.1007/s10103-021-03287-0, PMID: 33738615 PMC7972942

[17] Madani A Ahrari F Fallahrastegar A Daghestani N. A randomized clinical trial comparing the efficacy of low-level laser therapy (LLLT) and laser acupuncture therapy (LAT) in patients with temporomandibular disorders. Lasers Med Sci. (2020) 35:181–92. doi: 10.1007/s10103-019-02837-x, PMID: 31396794

[18] Yang HH Chung YC Szeto PP Yeh ML Lin JG. Laser acupuncture combined with auricular acupressure improves low-back pain and quality of life in nurses: a randomized controlled trial. J Integr Med. (2023) 21:26–33. doi: 10.1016/j.joim.2022.10.004, PMID: 36402666

[19] Willy RW Hoglund LT Barton CJ Bolgla LA Scalzitti DA Logerstedt DS. Patellofemoral pain: clinical practice guidelines linked to the international classification of functioning, disability and health from the academy of Orthopaedic physical therapy of the American Physical Therapy Association. J Orthop Sports Phys Ther. (2019) 49:CPG1–CPG95. doi: 10.2519/jospt.2019.0302 31475628

[20] Nouri F Raeissadat SA Eliaspour D Rayegani SM Rahimi MS Movahedi B. Efficacy of high-power laser in alleviating pain and improving function of patients with patellofemoral pain syndrome: a single-blind randomized controlled trial. J Lasers Med Sci. (2018) 10:37–43. doi: 10.15171/jlms.2019.06 31360367 PMC6499578

[21] Lehmann HC Wunderlich G Fink GR Sommer C. Diagnosis of peripheral neuropathy. Neurol Res Pract. (2020) 2:20. doi: 10.1186/s42466-020-00064-2, PMID: 33324924 PMC7650053

[22] Konin JG Lebsack D Valier A Isear JA Jr. Special tests for orthopedic examination. 4th ed. Boca Raton: CRC Press (2024).

[23] Motealleh A Mohamadi M Moghadam MB Nejati N Arjang N Ebrahimi N. Effects of Core neuromuscular training on pain, balance, and functional performance in women with patellofemoral pain syndrome: a clinical trial. J Chiropr Med. (2019) 18:9–18. doi: 10.1016/j.jcm.2018.07.006, PMID: 31193229 PMC6522640

[24] Vitoulas S Konstantis V Drizi I Vrouva S Koumantakis GA Sakellari V. The effect of physiotherapy interventions in the workplace through active Micro-break activities for employees with standing and sedentary work. Healthcare. (2022) 10:2073. doi: 10.3390/healthcare10102073, PMID: 36292520 PMC9603092

[25] Ozlu O Atilgan E. The effect of high-intensity laser therapy on pain and lower extremity function in patellofemoral pain syndrome: a single-blind randomized controlled trial. Lasers Med Sci. (2024) 39:103. doi: 10.1007/s10103-024-04017-y, PMID: 38630331 PMC11024020

[26] White A Cummings TM Filshie J. An introduction to Western medical acupunture. 2nd ed. Edinburgh, New York: Elsevier Saunders (2018). 234 p.

[27] Helianthi DR Simadibrata C Srilestari A Wahyudi ER Hidayat R. Pain reduction after laser acupuncture treatment in geriatric patients with knee osteoarthritis: a randomized controlled trial. Acta Med Indones. (2016) 48:114–21. 27550880

[28] Delgado DA Lambert BS Boutris N McCulloch PC Robbins AB Moreno MR. Validation of digital visual analog scale pain scoring with a traditional paper-based visual analog scale in adults. JAAOS Glob Res Rev. (2018) 2:e088. doi: 10.5435/JAAOSGlobal-D-17-00088, PMID: 30211382 PMC6132313

[29] Hancock GE Hepworth T Wembridge K. Accuracy and reliability of knee goniometry methods. J Exp Orthop. (2018) 5:46. doi: 10.1186/s40634-018-0161-5, PMID: 30341552 PMC6195503

[30] Shamsi M Mirzaei M Khabiri SS. Universal goniometer and electro-goniometer intra-examiner reliability in measuring the knee range of motion during active knee extension test in patients with chronic low back pain with short hamstring muscle. BMC Sports Sci Med Rehabil. (2019) 11:4. doi: 10.1186/s13102-019-0116-x, PMID: 30949343 PMC6431043

[31] Dos Santos RA Derhon V Brandalize M Brandalize D Rossi LP. Evaluation of knee range of motion: correlation between measurements using a universal goniometer and a smartphone goniometric application. J Bodyw Mov Ther. (2017) 21:699–703. doi: 10.1016/j.jbmt.2016.11.008, PMID: 28750987

[32] Haddad BI Hamdan M Isleem U Al-Saber MG Al-Hadidi FA AlRyalat SA. Validation of the cultural adaptation of the Kujala score in Arabic. J Orthop Surg Res. (2021) 16:323. doi: 10.1186/s13018-021-02489-0, PMID: 34011354 PMC8132389

[33] Chon TY Mallory MJ Yang J Bublitz SE Do A Dorsher PT. Laser acupuncture: a concise review. Med Acupunct. (2019) 31:164–8. doi: 10.1089/acu.2019.1343, PMID: 31297170 PMC6604908

[34] Dos Santos AF Nakagawa TH Lessi GC Luz BC Matsuo HTM Nakashima GY. Effects of three gait retraining techniques in runners with patellofemoral pain. Phys Ther Sport. (2019) 36:92–100. doi: 10.1016/j.ptsp.2019.01.006, PMID: 30703643

[35] Crossley KM Van Middelkoop M Callaghan MJ Collins NJ Rathleff MS Barton CJ. 2016 patellofemoral pain consensus statement from the 4th international patellofemoral pain research retreat, Manchester. Part 2: recommended physical interventions (exercise, taping, bracing, foot orthoses and combined interventions). Br J Sports Med. (2016) 50:844–52. doi: 10.1136/bjsports-2016-096268, PMID: 27247098 PMC4975825

[36] WALT recommendations. Available online at: https://waltza.co.za/documentation-links/recommendations (Accessed December 17, 2024). (2012).

[37] Bahr FR Litscher G. Laserakupunktur und innovative Lasermedizin. München: Elsevier, Urban & Fischer (2015).

[38] Cotler HB Chow RT Hamblin MR Carroll J. The use of low level laser therapy (LLLT) for musculoskeletal pain. MOJ Orthop Rheumatol. (2015) 2:0068. doi: 10.15406/mojor.2015.02.00068, PMID: 26858986 PMC4743666

[39] Crossley KM Bennell KL Cowan SM Green S. Analysis of outcome measures for persons with patellofemoral pain: which are reliable and valid?11No commercial party having a direct financial interest in the results of the research supporting this article has or will confer a benefit upon the author(s) or upon any organization with which the author(s) is/are associated. Arch Phys Med Rehabil. (2004) 85:815–22. doi: 10.1016/S0003-9993(03)00613-0, PMID: 15129407

[40] Bloom DA Kaplan DJ Mojica E Strauss EJ Gonzalez-Lomas G Campbell KA. The minimal clinically important difference: a review of clinical significance. Am J Sports Med. (2023) 51:520–4. doi: 10.1177/03635465211053869, PMID: 34854345

[41] Chen Z Ma C Xu L Wu Z He Y Xu K. Laser acupuncture for patients with knee osteoarthritis: a systematic review and Meta-analysis of randomized placebo-controlled trials. Evid Based Complement Alternat Med. (2019) 2019:1–10. doi: 10.1155/2019/6703828, PMID: 31781275 PMC6874873

[42] Al Rashoud AS Abboud RJ Wang W Wigderowitz C. Efficacy of low-level laser therapy applied at acupuncture points in knee osteoarthritis: a randomised double-blind comparative trial. Physiotherapy. (2014) 100:242–8. doi: 10.1016/j.physio.2013.09.007, PMID: 24418801

[43] Yetişir A Öztürk GY. Effects of low-level laser therapy on acupuncture points on knee pain and function in knee osteoarthritis. Rev Assoc Med Bras. (2024) 70:e20230264. doi: 10.1590/1806-9282.20230264, PMID: 38126411 PMC10740188

[44] Elgendy A Zikri EN Shafei HF Monir R Elwahidy AG Ali MA. Evaluation of the efficacy of gallium-aluminum-arsenide laser acupuncture in the management of knee osteoarthritis. J Arab Soc Med Res. (2021) 16:167–72. doi: 10.4103/jasmr.jasmr_29_21

[45] Lafta Mezaal A Bashardoust Tajali S Olyaei G Jalaie S Thweab AS. Effects of low-level laser versus laser acupuncture in patients with knee osteoarthritis: a randomized controlled trial. J Mod Rehabil. (2019) 30:181–94. doi: 10.32598/JMR.V12.N3.181

[46] Stausholm MB Naterstad IF Joensen J Lopes-Martins RÁB Sæbø H Lund H. Efficacy of low-level laser therapy on pain and disability in knee osteoarthritis: systematic review and meta-analysis of randomised placebo-controlled trials. BMJ Open. (2019) 9:e031142. doi: 10.1136/bmjopen-2019-031142, PMID: 31662383 PMC6830679

[47] Yurtkuran M Alp A Konur S Özçakir S Bingol U. Laser acupuncture in knee osteoarthritis: a double-blind, randomized controlled study. Photomed Laser Surg. (2007) 25:14–20. doi: 10.1089/pho.2006.1093, PMID: 17352632

[48] Silva MDC Woodward AP Fearon AM Perriman DM Spencer TJ Couldrick JM. Minimal clinically important change of knee flexion in people with knee osteoarthritis after non-surgical interventions using a meta-analytical approach. Syst Rev. (2024) 13:50. doi: 10.1186/s13643-023-02393-0, PMID: 38303000 PMC10832130

[49] Guzik A Drużbicki M Wolan-Nieroda A Turolla A Kiper P. Estimating minimal clinically important differences for knee range of motion after stroke. J Clin Med. (2020) 9:3305. doi: 10.3390/jcm9103305, PMID: 33076214 PMC7602397

[50] Rayegani SM Raeissadat SA Heidari S Moradi-Joo M. Safety and effectiveness of low-level laser therapy in patients with knee osteoarthritis: a systematic review and Meta-analysis. J Lasers Med Sci. (2017) 8:S12–9. doi: 10.15171/jlms.2017.s3, PMID: 29071029 PMC5642172

[51] Tim CR Bossini PS Kido HW Malavazi I Von Zeska Kress MR Carazzolle MF. Low-level laser therapy induces an upregulation of collagen gene expression during the initial process of bone healing: a microarray analysis. J Biomed Opt. (2016) 21:088001. doi: 10.1117/1.JBO.21.8.088001, PMID: 27548776

[52] Alghamdi BA Karkousha RN Elgeidi AA Amin FS Tolba AM. Effect of low-level laser therapy on knee range of motion and functional abilities after total knee arthroplasty. Cureus. (2023) 15:e50893. doi: 10.7759/cureus.50893 38249281 PMC10799633

[53] Li C Chen Y Lin T Hsiao Y Fu JC Chen C. Immediate responses of multi-focal low level laser therapy on quadriceps in knee osteoarthritis patients. Kaohsiung J Med Sci. (2019) 35:702–7. doi: 10.1002/kjm2.12113, PMID: 31390143 PMC11900728

[54] Jankaew A You YL Yang TH Chang YW Lin CF. The effects of low-level laser therapy on muscle strength and functional outcomes in individuals with knee osteoarthritis: a double-blinded randomized controlled trial. Sci Rep. (2023) 13:165. doi: 10.1038/s41598-022-26553-9, PMID: 36599881 PMC9812996

[55] Huang CH Yeh ML Chen FP Kuo M. A randomised controlled trial of laser acupuncture improves early outcomes of osteoarthritis patients’ physical functional ability after total knee replacement. Complement Ther Clin Pract. (2021) 43:101340. doi: 10.1016/j.ctcp.2021.101340, PMID: 33677172

[56] Santinoni CS Neves APC Almeida BFM Kajimoto NC Pola NM Caliente EA. Bone marrow coagulated and low-level laser therapy accelerate bone healing by enhancing angiogenesis, cell proliferation, osteoblast differentiation, and mineralization. J Biomed Mater Res A. (2021) 109:849–58. doi: 10.1002/jbm.a.37076, PMID: 32815657

[57] Melo MDO Pompeo KD Baroni BM Sonda FC Vaz MA. Randomised study of the effects of neuromuscular electrical stimulation and low-level laser therapy on muscle activation and pain in patients with knee osteoarthritis. Int J Ther Rehabil. (2019) 26:1–13. doi: 10.12968/ijtr.2018.0089

[58] Hung YC Lin PY Chiu HE Huang PY Hu WL. The effectiveness of laser acupuncture for treatment of musculoskeletal pain: a Meta-analysis of randomized controlled studies. J Pain Res. (2021) 14:1707–19. doi: 10.2147/JPR.S308876, PMID: 34163229 PMC8214113

[59] Han R Guo C Lau K Hu J. Efficacy of knee osteoarthritis by use of laser acupuncture: a systematic review and meta-analysis. Medicine. (2024) 103:e38325. doi: 10.1097/MD.0000000000038325, PMID: 38905420 PMC11191916

[60] Youssef EF Muaidi QI Shanb AA. Effect of laser therapy on chronic osteoarthritis of the knee in older subjects. J Lasers Med Sci. (2016) 7:112–9. doi: 10.15171/jlms.2016.19, PMID: 27330707 PMC4909009

[61] Kim GJ Choi J Lee S Jeon C Lee K. The effects of high intensity laser therapy on pain and function in patients with knee osteoarthritis. J Phys Ther Sci. (2016) 28:3197–9. doi: 10.1589/jpts.28.3197, PMID: 27942148 PMC5140828

[62] Sharif F Shoukat H Arif MA. Effects of strengthening of hip abductors and lateral rotators for improving pain and functional limitation in patients with patellofemoral dysfunction. Rawal Med J. (2020) 45:236–9.

[63] Robbins SR Alfredo PP Junior WS Marques AP. Low-level laser therapy and static stretching exercises for patients with knee osteoarthritis: a randomised controlled trial. Clin Rehabil. (2022) 36:204–13. doi: 10.1177/02692155211047017, PMID: 34714175

