# Supplementary material for: Effect of laser acupuncture on pain, range of motion, and function in patellofemoral pain syndrome: a randomised controlled trial
Source: Front Med (Lausanne). 2025 Jul 15;12:1613197. doi: 10.3389/fmed.2025.1613197 (PMC12303989; doi:10.3389/fmed.2025.1613197)
Supplement: SUPPLEMENTARY TABLE S1 — Description of traditional physical therapy program used in the study. [file Table_1.docx]

**Table S1: Description of traditional physical therapy program used in the study.**

| **Exercise** | **Description** | **Duration and Repetitions** |
| --- | --- | --- |
| **Isometric quadriceps exercises** | The participant was asked to sit at the edge of the bed, a rolled-up towel was placed under the knee while placing it at the edge of the bed. Then, the elastic Thera-band was placed at bedpost underneath the foot. The participant held the knee in full extension. | Hold 5-10 seconds, 3 sets of 10 repetitions. |
| **Isometric hip adduction** | The participant was instructed to stand next to the bed. The Thera-band was tied to a bedpost, and the participant placed a Thera-band around the thigh just above the knee of the exercised leg. Gently press the knee inward without moving the feet, the participant held position. | Hold 5-10 seconds, 3 sets of 10 repetitions. |
| **Straight leg raising (upward)** | Participant was asked to lie supine on the bed, keep the affected leg straight and the other leg bent at the knee with the foot flat on the bed for support. Then, tighten the quadriceps muscle of the straight leg so that the knee is fully extended. Slowly lift the straight leg up to approximately **45 degrees** (or about the height of the opposite bent knee). **Hold** the position for **5–10 seconds**. Then, slowly lower the leg back to the bed. | Hold 5-10 seconds, 3 sets of 10 repetitions. |
| **Straight leg raising (laterally)** | Participant was instructed to lie on his/ her side on a mat/ bed. The **bottom leg (**non-affected or less affected) can be slightly bent for stability, while keeping the **top leg (affected) straight**, aligned with the trunk and hips stacked vertically. Rest the head on a pillow for neck support. Place the top hand on the mat for balance. Then, slowly raise the **top leg upward** about 30–45 degrees from the bed, keeping it **straight** and in line with the body (not swinging forward). Hold at the top**.** Slowly **lower** the leg back down to the starting position in a controlled motion. | Hold 5-10 seconds, 3 sets of 10 repetitions. |
| **Squat to 30° knee flexion** | Participant was asked to stand against the wall with a gymnastic ball in the middle of the back with feet facing away from the wall. Then, squat down slowly on the ball till 30° knee flexion and moves slowly back up. | Hold 10-15 seconds, repeat 5-10 times. |
| **Stretching exercises** | **1. Hamstring muscle:**  From sitting position, the participant was asked to bend the knee of the unaffected or less affected leg and keep the affected leg extended with the knee slightly bent. Bend at the waist towards the foot. Hold the lower leg for support.  **2. Gastrocnemius:**  From standing position, the participant was instructed to put both hands against the wall. Keep back leg straight. Then, push heels down and slowly lean forward until a stretch is felt in the back of the calf.  **3. Iliotibial band:**  The participant was asked to lie on the back with both legs straight. Lift the affected **leg up** and cross it over the non- affected or less affected leg, letting it fall toward the floor. Keep the **shoulders flat** on the ground. Use the opposite hand to gently pull the affected leg down for a deeper stretch until feeling the stretch along the **outside of the affected leg.** | Hold for 10 – 15 seconds Repeat 2-3 times. |
